# Habits Heart App for Patient Engagement in Heart Failure Management: Pilot Feasibility Randomized Trial

**DOI:** 10.2196/19465

**Published:** 2021-01-20

**Authors:** Kevin S Wei, Nasrien E Ibrahim, Ashok A Kumar, Sidhant Jena, Veronica Chew, Michal Depa, Namrata Mayanil, Joseph C Kvedar, Hanna K Gaggin

**Affiliations:** 1 Cardiology Division Massachusetts General Hospital Boston, MA United States; 2 University of California, Irvine, School of Medicine Irvine, CA United States; 3 Harvard Medical School Boston, MA United States; 4 Jana Care Boston, MA United States; 5 Department of Dermatology Massachusetts General Hospital Boston, MA United States

**Keywords:** heart failure, smartphone application, heart failure management

## Abstract

**Background:**

Due to the complexity and chronicity of heart failure, engaging yet simple patient self-management tools are needed.

**Objective:**

This study aimed to assess the feasibility and patient engagement with a smartphone app designed for heart failure.

**Methods:**

Patients with heart failure were randomized to intervention (smartphone with the Habits Heart App installed and Bluetooth-linked scale) or control (paper education material) groups. All intervention group patients were interviewed and monitored closely for app feasibility while receiving standard of care heart failure management by cardiologists. The Atlanta Heart Failure Knowledge Test, a quality of life survey (Kansas City Cardiomyopathy Questionnaire), and weight were assessed at baseline and final visits.

**Results:**

Patients (N=28 patients; intervention: n=15; control: n=13) with heart failure (with reduced ejection fraction: 15/28, 54%; male: 20/28, 71%, female: 8/28, 29%; median age 63 years) were enrolled, and 82% of patients (N=23; intervention: 12/15, 80%; control: 11/13, 85%) completed both baseline and final visits (median follow up 60 days). In the intervention group, 2 out of the 12 patients who completed the study did not use the app after study onboarding due to illnesses and hospitalizations. Of the remaining 10 patients who used the app, 5 patients logged ≥1 interaction with the app per day on average, and 2 patients logged an interaction with the app every other day on average. The intervention group averaged 403 screen views (per patient) in 56 distinct sessions, 5-minute session durations, and 22 weight entries per patient. There was a direct correlation between duration of app use and improvement in heart failure knowledge (Atlanta Heart Failure Knowledge Test score; ρ=0.59, *P*=.04) and quality of life (Kansas City Cardiomyopathy Questionnaire score; ρ=0.63, *P*=.03). The correlation between app use and weight change was ρ=–0.40 (*P*=.19). Only 1 out of 11 patients in the control group retained education material by the follow-up visit.

**Conclusions:**

The Habits Heart App with a Bluetooth-linked scale is a feasible way to engage patients in heart failure management, and barriers to app engagement were identified. A larger multicenter study may be warranted to evaluate the effectiveness of the app.

**Trial Registration:**

ClinicalTrials.gov NCT03238729; http://clinicaltrials.gov/ct2/show/NCT03238729

## Introduction

The COVID-19 pandemic has disrupted chronic disease management [1]. In particular, patients with heart failure are at high risk of complications from disruption of care. Heart failure management is complex; it requires taking multiple medications, a close monitoring of total daily fluid and sodium intake, and monitoring for volume and symptoms. Clinical changes may warrant timely intervention in medication regimen to avoid life-threatening complications. Despite the importance of adherence to medical recommendations, in patients living with heart failure, patient engagement, adherence, and self-management are suboptimal and may limit the benefits of heart failure recommendations and existing treatments [2-4]. Smartphones and mobile health apps are ubiquitous even in older adult populations [5,6]. Smartphones and mobile health apps have the potential for bettering patient engagement and self-management. While there are a number of commercially available smartphone heart failure apps, few have been designed by clinicians or specifically designed to address heart failure self-management; only 41% had weight management features [7]. Even fewer have published their methodology and feasibility studies [8,9] in peer-reviewed journals.

In this context, we employed a heart failure–specific smartphone-based app intervention with content designed by clinicians with the following features: daily to-do lists for heart failure–related activities including patient education, symptom monitoring, wireless weight tracking, sodium intake tracking, exercise logs, and action prompts based on weight and symptom severity. There was also a messaging platform for patients to reach out to study staff and physicians. Our goal was to test feasibility and usability in this pilot randomized proof-of-concept study.

## Methods

All study procedures were approved by the Partners Healthcare Institutional Review Board and carried out in accordance with the Declaration of Helsinki, and the study was registered (NCT03238729). The Habits Heart App was developed in conjunction with cardiologists who care for patients with heart failure at Massachusetts General Hospital and software engineers at Jana Care. Cardiologists designed the main concepts (daily To-Do list, Learn, Track, Coach) and content in writing and worked with Jana Care to develop the app. While the main goal of Track was to monitor weight changes, tracking sodium content (in diet) and exercising logging were also included, in an exploratory fashion, using a publicly available database of exercises and a database from University of Minnesota of the sodium content in food items. The Habits Heart App was downloaded onto a study Android smartphone.

**Figure 1:**
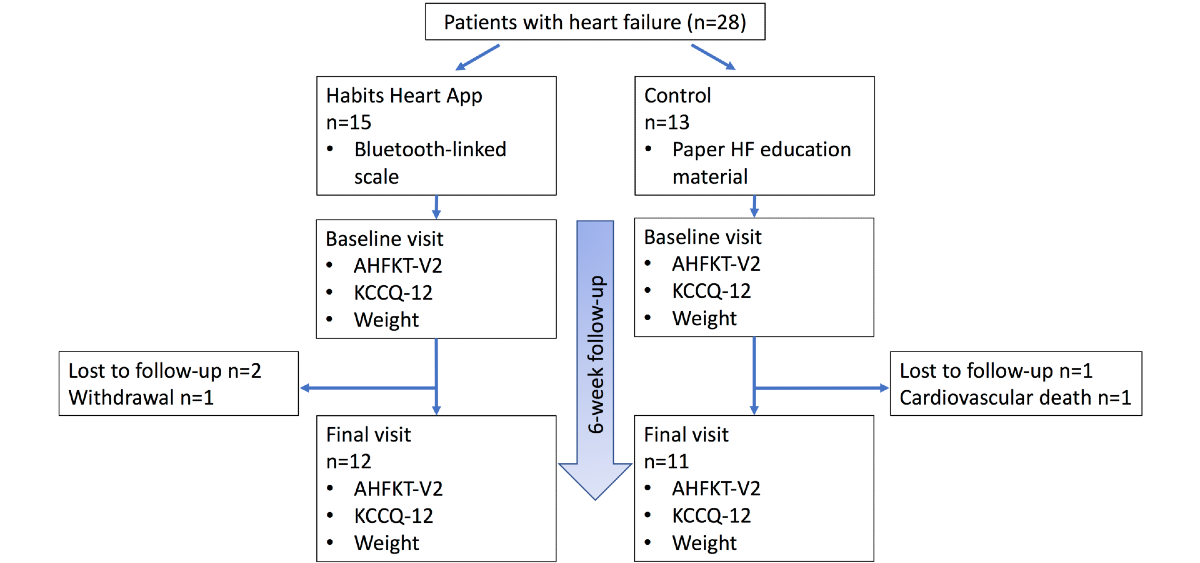
Study flow diagram. AHFKT-V2: Atlanta HF Knowledge Test, Version 2; HF: heart failure; KCCQ-12: Kansas City Cardiomyopathy Questionnaire.

Patients with heart failure aged ≥18 years with smartphones were recruited from cardiology clinics and inpatient units at Massachusetts General Hospital. Heart failure diagnosis by a cardiologist in the electronic medical records with ≥1 hospitalization or emergency department visit for acute heart failure or any outpatient escalation of diuretic therapy in the past 12 months was required. *Heart failure with reduced ejection fraction* was defined as symptomatic heart failure with a left ventricular ejection fraction ≤40%, and *heart failure with preserved ejection fraction* was defined as symptomatic heart failure with left ventricular ejection fraction ≥50% by any imaging modality. A randomized list with the order of intervention or control group allocations corresponding to the order of enrollment was generated at the start of the study. After informed consent was obtained, patients were then allocated to either the intervention group or the control group (Figure 1). Those randomized to the intervention group were given a 30-minute onboarding session to get acquainted with the Habits Heart App and the study smartphone. At the end of the 30-minute session, patients were asked to demonstrate their app use competency by performing actions in 5 key areas including opening and watching the introduction patient education video, logging weight on the app by using the Bluetooth-linked scale, inputting sodium content entries, inputting exercise entries, and messaging the study team through the app.

All patients received the standard of care for heart failure management by cardiologists. Patients in the control group received written heart failure education materials covering the same topics as the patient education portion of the app and were instructed to read the patient education materials until the final visit.

**Figure 2:**
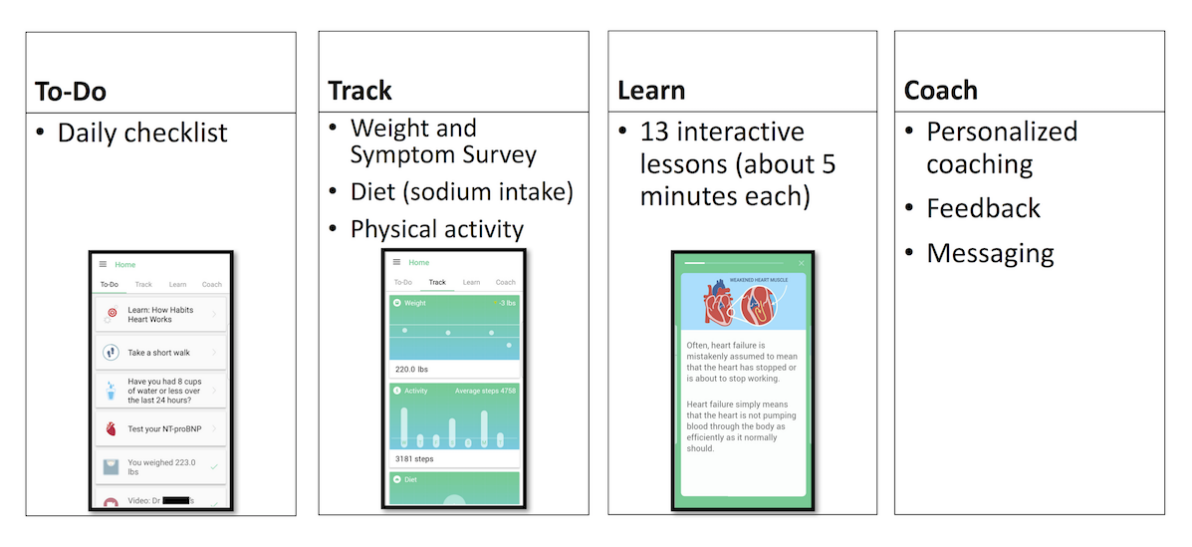
Layout of the Habits Heart App: To-Do List, Track, Learn, Coach.

Intervention group patients were provided a smartphone with the Habits Heart App. The daily To-Do list prompted patients to watch the educational heart failure video of the day, exercise, weigh themselves, or complete other healthy activities in addition to 3 self-management functionalities: Track, Learn, and Coach (Figure 2). Track synced automatically with a Bluetooth-linked digital scale to record daily weight. Weight gain ≥3 lbs (1.4 kg) in 1 to 2 days or ≥5 lbs (2.3 kg) in a week triggered a symptoms survey. Depending on symptom severity, the app had an automated risk-based algorithm to instruct patients to contact study staff or contact their doctor (or dial 911, ie, emergency services, if after hours). If patients were instructed to call a physician or dial 911, the study team would follow up with patients by phone within 1 day if during the week or on Monday of the next week if during the weekend. Diet and physical activities were tracked in an exploratory fashion. Patients picked foods consumed at each meal from a list of available food items with corresponding sodium content, while patients entered length and mode of exercise performed each day. The exercise database had not previously been validated. Learn featured 13 interactive lessons including videos with topics recorded and narrated by cardiologists. Coach included a messaging platform where patients could communicate with the study team and cardiologist. All patients in the intervention group were contacted through the app messaging platform by the study staff within 1 week of starting the study to obtain feasibility feedback regarding the study app. The study coordinator tracked each patient’s messages sent to the study team along with weight, sodium content, and exercise entries through a secure online dashboard with deidentified patient data accessible only to the study staff. At the final study visit, all patients were interviewed for feasibility feedback.

Baseline characteristics were obtained from patient electronic medical records. At baseline and final visits (goal of at least 6 weeks in-between), the Atlanta Heart Failure Knowledge Test (AHFKT-V2) [10] and a quality of life survey (Kansas City Cardiomyopathy Questionnaire, KCCQ-12) [11] were filled out by patients along with recording of weight for both intervention and control groups. The KCCQ-12 scores were normalized to a 0-to-100-point scale. Higher AHFKT-V2 or KCCQ-12 scores indicated better heart failure-specific education or health status. Patients who did not have hospital follow-up visits already scheduled 6 weeks after study onboarding were asked to schedule an appointment for the earliest availability after 6 weeks. Intervention group patients were asked to return study smartphones and Bluetooth scales upon study completion. Control group patients were asked if they retained paper patient education material.

Spearman correlations were used to determine the relationships between the average session duration of app use and the AHFKT-V2 score, the KCCQ-12 score, and weight loss.

## Results

### Patient Characteristics

A total of 28 patients were enrolled in the study (intervention: n=15; control: n=13). Of the patients enrolled, 54% (15/28) had heart failure with reduced ejection fraction, 71% (20/28) were male, and the median age was 63 years. The median follow-up was 60 days (range 50-66 days); 80% of patients (12/15) in the intervention and 85% of patients (11/13) in the control group completed both baseline and final visits (Table 1). In the intervention group, 2 patients were lost to follow-up, and 1 patient withdrew from the study. In the control group, 1 patient was lost to follow-up, and 1 patient died.

**Table 1:** Baseline characteristics of patients with heart failure.

Characteristics			App (n=12)	Control (n=11)
**Demographic**				
	Age (years), median		63	62
	**Sex, n (%)**			
		Male	9 (75)	7 (64)
		Female	3 (25)	4 (36)
	**Race, n (%)**			
		African-American	2 (17)	0 (0)
		Asian	1 (8)	0 (0)
		Caucasian	8 (67)	11 (100)
		Hispanic	1 (8)	0 (0)
	Weight (kg), median		98.1	93.4
	With reduced ejection fraction, n (%)		5 (42)	6 (55)
	**New York Heart Association class^a^, n (%)**			
		Class I	3 (33)	1 (13)
		Class II	6 (67)	3 (38)
		Class III	0 (0)	3 (38)
		Class IV	0 (0)	1 (13)
**Medical history, n (%)**				
	Atrial fibrillation/flutter		8 (67)	2 (18)
	Hypertension		12 (100)	9 (82)
	Coronary artery disease		9 (75)	5 (45)
	Chronic obstructive pulmonary disease		2 (17)	0 (0)
	Type 1 or type 2 diabetes		6 (50)	5 (45)
	Hyperlipidemia		9 (75)	8 (73)
	Cerebrovascular accident/transient ischemic attack		4 (33)	0 (0)
	Chronic kidney disease		6 (50)	3 (27)
**Medication, n (%)**				
	Angiotensin converting enzyme inhibitor		3 (25)	3 (27)
	Angiotensin II receptor blocker		3 (25)	4 (36)
	Sacubitril/valsartan		4 (33)	1 (9)
	Beta blocker		11 (92)	9 (82)
	Mineralocorticoid receptor antagonist		5 (42)	3 (27)
	Loop diuretics		12 (100)	10 (91)
**Echocardiography results**				
	Left ventricular ejection fraction (%)		47.4	40.9
**Biomarkers, median**				
	N-terminal pro–B-type natriuretic peptide (pg/mL)		669	1910

^a^App: n=9; control: n=8. Note: As a result of rounding, percentages may not add to 100.

### Intervention Feasibility

**Figure 3:**
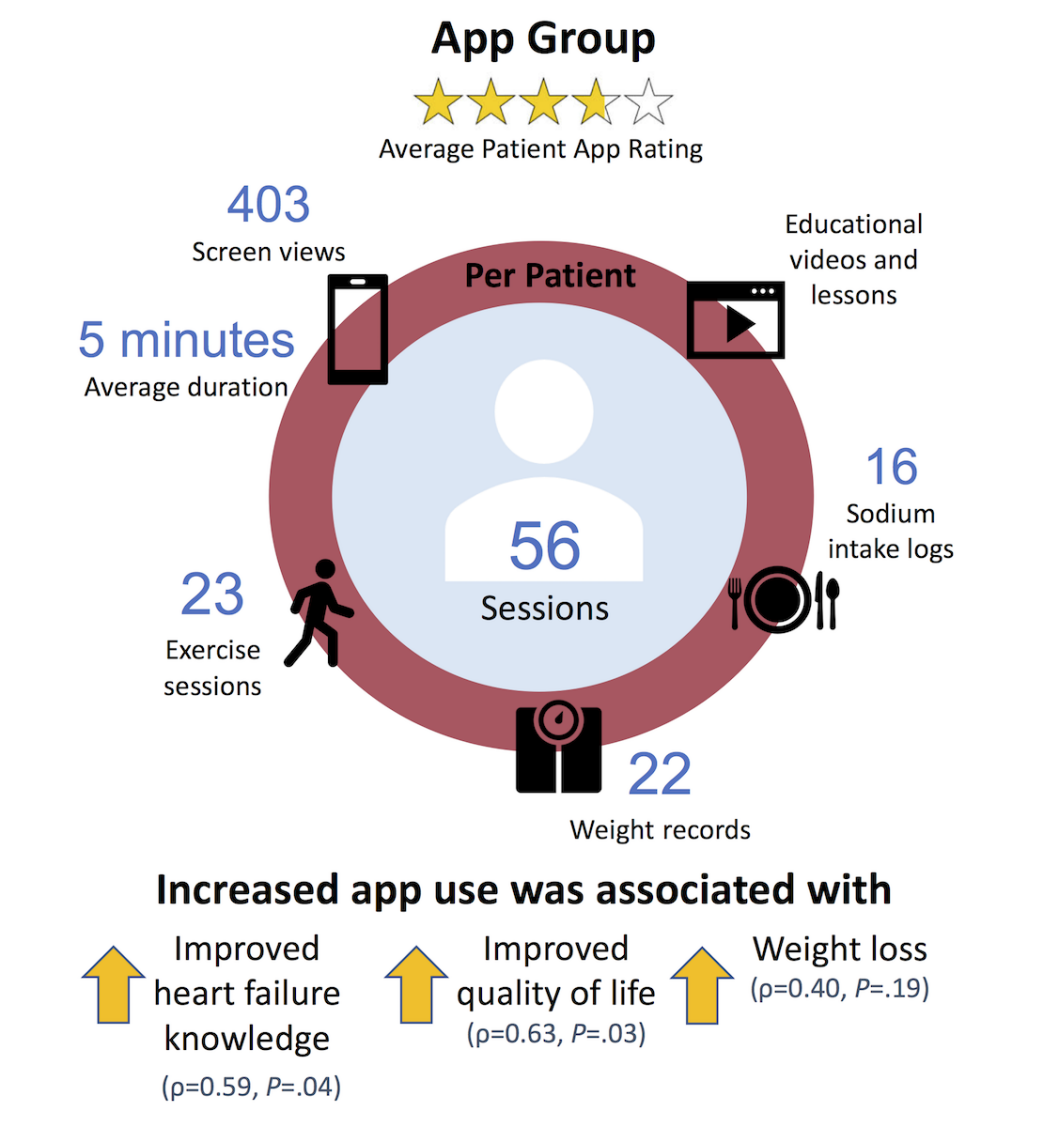
App feasibility and patient rating.

All patients in the intervention group (15/15, 100%) were able to demonstrate their competency using the app by performing actions in 5 key areas at the end of the 30-minute onboarding session; however, 2 out of the 12 patients (17%) who completed both study visits were unable to use the app after study onboarding due to intermittent illnesses and hospitalizations. Of the 10 patients who were able to use the app, 7 patients (70%) logged ≥1 interaction with the app every other day on average throughout the study duration, and 5 patients (50%) logged ≥1 interaction with the app per day on average throughout the study duration. At the final visit, 9 out of 12 patients (75%) in the intervention group subjectively reported regularly interacting with the app (Figure 3).

There was an average of 403 screen views per person over 56 distinct sessions (duration per session, average: 5 minutes; documentation per person, average—weight: 22; sodium intake: 16; exercise: 23). The patients who reported using the app regularly (n=9) averaged 527 screen views per person over 73 distinct sessions (duration per session, average: 5 minutes; documentation per person, average—weight: 29; sodium intake: 21; exercise: 30).

As of the 6-week follow-up visit, 0 out of 12 patients had problems opening and watching education sessions on Learn. Only 1 out of 12 patients (8%) reported synching issues between the scale and the app, 1 out of 12 patients (8%) reported not being able to send additional messages on the app messaging platform for several minutes after sending the first message, and 2 out of 12 patients (17%) had problems with the smartphone intermittently disconnecting from the internet. There were 28 app crashes among the entire group. Only 1 out of 11 patients (9%) in the control group retained reading materials by the follow-up visit.

Patients rated the app (average 3.8 out of 5) and described enjoying the app’s educational lessons and videos most, followed by the weight recording feature, and the messaging platform. Patients reported that sometimes they could not find the exact food that they had consumed in the diet database, so they input the next-best option. Throughout the study, 48 symptoms were logged by app users, of which 25 triggered a prompt to contact the study team, and 4 required that patients call their doctors or call 911. In total, 11 messages were sent by app users to the study coordinator through the messaging platform regarding symptoms and trouble-shooting of the app.

### App Use and Heart Failure Education, Quality of Life, and Weight

The more a patient interacted with the app (higher the average session duration), the greater the improvement in AHFKT-V2 (ρ=0.59, *P=*.04) and KCCQ-12 (ρ=0.63, *P=*.03) scores from baseline to follow-up (Figure 3). The correlation between app use and weight loss was ρ=–0.40 (*P*=.19).

The median AHFKT-V2 score was 80% at both baseline and final visits for the control arm while the median score was 83.3% at baseline and 85% at the final visit for the intervention arm. The median KCCQ-12 score was 55 at baseline and 41 at the final visit for the control arm, while the median score was 74 at baseline and 70 at the final visit for the intervention arm. Patients in the control arm gained 2.5 lbs (1.1 kg) bodyweight during the study while patients in the app arm lost 3.8 lbs (1.7 kg) bodyweight.

## Discussion

In this pilot proof-of-concept study in a sample of patients with heart failure, we found that the Habits Heart App with a Bluetooth-linked scale was feasible and easily implemented. All of the patients in the intervention group were able to demonstrate that they could use the 5 key areas of the app on their own after a 30-minute onboarding session, and almost all of the patients were able to use the app without major stability, performance, or standards adherence issues. The Habits Heart App with scale is unique in that it was designed by clinicians who care for patients with heart failure to address a patient’s complex self-management needs for patient education, tracking, and coaching without requiring resource-intensive telemonitoring. While intensive monitoring may help to decrease adverse events in patients with heart failure, telemonitoring can require a lot of resources and be financially taxing [12]. Resource-intensive heart failure management may not be practical for the vast majority of patients with heart failure and may be best suited for high-risk patients [13]. As our experience has shown (patients with frequent hospitalizations not being able to use the app consistently), lower risk patients with heart failure may benefit from the Habits Heart App. Compared with a real-life control group, intervention group patients appeared to have better heart failure knowledge, quality of life, and weight. We also showed that greater involvement with the intervention was associated with greater improvement in heart failure knowledge and quality of life and may be associated with weight loss rather than gain over time.

Compared with a previously published study [8], our control group closely resembled the situation of real-life patients with heart failure; Athilingam and colleagues [8] used an active wait-listed control group with patients who were given an app with heart failure education features that incentivized the patients with the activation of additional features upon reaching the end of the study, which may have motivated patients in the control group to engage more than they typically would in real life. On a separate note, only 1 out of 11 control patients (9%) in our study retained patient reading materials by the follow-up visit. This statistic supports the notion that the usual standard of care of reading materials and handouts may not be engaging for patients with heart failure [14]. Heart failure education delivered through an app, such as the Habits Heart App, would be at the patients’ (literal) fingertips and has the potential to adapt to the individual’s learning level or style.

While there are several patient-centered heart failure apps available, few have been designed by clinicians who care for patients with heart failure or have been evaluated with a scientific assessment or publication in a medical journal to date [8,9,15-17]. Several well-designed, previously published feasibility studies utilized a more resource-intensive telemonitoring approach (such as a chest strap that monitors physiological data and accelerometer or blood pressure monitoring) in combination with a smartphone app [8,9]. In our study, we focused on the essentials that would not require intensive monitoring or devices for the patient or health care staff. We believe that such an approach would be more easily applied to broad clinical practice where resources may be scarce. The Habits Heart App features a comprehensive interactive tool for patients with heart failure that allows tracking of healthy habits, education of heart failure disease management, and direct contact with study staff and cardiologists for questions regarding app use or medical symptoms. Additionally, the Bluetooth-linked scale allows patients to measure weight directly and visualize trends in a centralized setting. Having customizable, prespecified automatic triggers for involving patients and notifying clinicians (rather than feeding raw data) may reduce the burden on already busy clinicians while empowering patients.

There are several areas identified in the feasibility assessment to address for the future iteration of the app including Bluetooth connectivity with the app, messenger delays, and sodium and exercise logging issues. Enabling app download onto patients’ own smartphones, whether iPhone or Android, will solve the internet connectivity issue and decrease the inconvenience of carrying 2 smartphones. An ability to sync summary data with electronic health records may further reduce administrative burden. Addition of mobile laboratory capabilities to check B-type natriuretic peptides, N-terminal pro–B-type natriuretic peptides, and basic metabolic panel may expand the utility of the app.

Despite randomization, there were differences in patient characteristics between the app and control groups, likely due to the small sample size of this feasibility study. The control group appeared to have more advanced heart failure (higher proportion of patients with New York Heart Association Class III or IV and higher N-terminal pro–B-type natriuretic peptide values), while the intervention group had a higher burden of comorbidities such as atrial fibrillation, coronary artery disease, chronic obstructive pulmonary disease, and chronic kidney disease. The differences between the two groups may potentially result in more improvement in the app group compared with that in the control group; patients with less advanced heart failure may score better. However, in order to minimize the potential impact of baseline differences in the two groups, we looked at the relative change in heart failure knowledge, quality of life, and weight loss rather than the final or absolute values. Nonetheless, any definitive conclusions about the app’s effects on quality of life or heart failure knowledge should be evaluated with a large multicenter trial.

The Habits Heart App is a feasible way to dynamically engage patients in heart failure management and equip patients with effective self-management tools to potentially improve heart failure knowledge, quality of life, and adherence to medical recommendations. A larger multicenter trial is needed to further test the use of the app and clinical outcomes.

## Appendix

Multimedia Appendix 1 CONSORT-eHEALTH checklist (V 1.6.1).

## Abbreviations

AHFKT-V2: Atlanta Heart Failure Knowledge Test, Version 2
KCCQ-12: Kansas City Cardiomyopathy Questionnaire
